# Transcriptional changes during ovule development in two genotypes of litchi (*Litchi chinensis* Sonn.) with contrast in seed size

**DOI:** 10.1038/srep36304

**Published:** 2016-11-08

**Authors:** Ashish K. Pathak, Sudhir P. Singh, Yogesh Gupta, Anoop K. S. Gurjar, Shrikant S. Mantri, Rakesh Tuli

**Affiliations:** 1 National Agri-Food Biotechnology Institute, Department of Biotechnology, Mohali, Punjab, India; 2 Department of Biotechnology, Panjab University, Chandigarh-160014, India; 3 Center of Innovative and Applied Bioprocessing, Department of Biotechnology, Mohali, India; 4 University Institute of Engineering and Technology, Panjab University, Chandigarh, India

## Abstract

*Litchi chinensis* is a subtropical fruit crop, popular for its nutritional value and taste. Fruits with small seed size and thick aril are desirable in litchi. To gain molecular insight into gene expression that leads to the reduction in the size of seed in *Litchi chinensis*, transcriptomes of two genetically closely related genotypes, with contrasting seed size were compared in developing ovules. The cDNA library constructed from early developmental stages of ovules (0, 6, and 14 days after anthesis) of bold- and small-seeded litchi genotypes yielded 303,778,968 high quality paired-end reads. These were *de-novo* assembled into 1,19,939 transcripts with an average length of 865 bp. A total of 10,186 transcripts with contrast in expression were identified in developing ovules between the small- and large- seeded genotypes. A majority of these differences were present in ovules before anthesis, thus suggesting the role of maternal factors in seed development. A number of transcripts indicative of metabolic stress, expressed at higher level in the small seeded genotype. Several differentially expressed transcripts identified in such ovules showed homology with *Arabidopsis* genes associated with different stages of ovule development and embryogenesis.

*Litchi chinensis* Sonn. is a subtropical evergreen fruit tree of family Sapindaceae and sub family Nepheleae. Sapindaceae family comprises of about 150 genera and 2,000 species. The genus *Litchi* has three subspecies: *Litchi chinensis ssp. chinensis*, *philippinensis* and *javensis*. The subspecies *chinensis* bears arillate fruit, unlike long thorny protuberances with inedible flesh in ssp *philippinensisis* and thinner aril in ssp *javensis*. The genus originated in China, where it has been cultivated for more than 2,300 years.

Three types of flowers are found in litchi, type I staminate flowers, type II hermaphrodite female flowers and type III hermaphrodite flowers. Litchi fruits develop from bicarpellary ovary of type II hermaphrodite female flowers. Generally, only one carpel of type 2 flower develops into fruit. Each ovary contains an anatropous ovule, which develops into a seed. The edible part of the fruit, called aril, develops from the outer integument of ovule^1^. Botanically, the fruit is a drupe. Aril is sweet in taste, rich in calories, vitamin C, and is a good source of minerals^2^.

In angiosperms, seed development begins with double fertilization followed by the formation of embryo and endosperm. Endosperm development progresses through four phases: syncytial, cellularization, differentiation, and death^3^. In most dicots, initially the endosperm grows rapidly and is consumed at later stages. Thus, the cotyledon occupies most of the portion in a mature seed. Coordination in the growth of endosperm and embryo is instrumental in determining seed size in a fruit^4^. The size of seed is also limited by the expansion space permitted by seed coat. Some genes have been identified to play role in determining seed size in *Arabidopsis thaliana*^5^,^6^. However, no such studies have been reported on litchi.

Next generation sequencing technologies (NGS) and differential expression analyses have accelerated developmental studies in plants through the investigation of transcriptomes. In horticultural crops, only in a few cases, transcriptome sequence analysis has been used for the prediction of genes related to fruit development- as in case of *Annona squamosa, Musa accuminata*, *Prunusavium*, *Pyrusbrets chneideri* etc^7^,^8^,^9^,^10^. Comparative analysis of transcriptome in litchi has been used to speculate on the processes involved in the regulation of floral initiation by phytohormones, expression of flowering related genes^11^, genes related to shading stress^12^, fruit cracking^13^, reactive oxygen species (ROS) induced abortion of rudimentary leaves^14^, fruit abscission^15^, maturation, coloration^16^, abscission induced by carbohydrate stress^17^ and fruit ripening after cold storage^18^. To the best of our knowledge, there is no report on transcription profiling of early stage developing ovules in litchi.

Litchi seeds are very hard and bold, therefore not favourable to fruit juice processing industry and fresh fruit consumers. Small seed size or seedlessness is desirable in litchi, also because small-seeded fruits develop more pulp as compared to the bold-seeded genotypes^19^. Molecular characterization of seed development in ovule in genotypes with contrasting seed size may give leads to control seed development in litchi. In this study, we compared the transcriptional profiles in early-stage developing ovules of two litchi genotypes with contrast in seed size. The transcriptomes were assembled, annotated and analyzed for differential expression.

## Results and Discussion

### Sequence assembly and annotation

Transcriptome sequencing of litchi (bold and small seeded) ovule-specific libraries at 0, 6 and 14 DAA yielded 303,778,968 high-quality paired-end reads of average 101 nucleotides length. The reads were assembled into 1,19,939 transcripts (Trinity transcripts), with an average length of 865.38, median length of 489 and N50 length of 1,487. The 1,19,939 transcripts represented 87,072 unique components (unigenes) and their isoforms (Supplementary Table S1). Our study records the highest number of transcripts in litchi, reported till date.

The assembled transcripts were compared against the public databases (*E*-value ≤ 10^−5^). Out of the 1,19,939 transcripts, 48,968 (40.82%), 36,570 (30.49%) and 16,140 (13.45%) showed homology with the sequences in NCBI non-redundant (NR), Swiss-Prot and COG(Clusters of Orthologous Groups) databases, respectively. Comparative analysis was done with a few closely related species (*Citrus sinensis, Populus trichocarpa, Ricinus communis, Glycine max, Fragaria vesca, Carica papaya and Vitis vinifera*) and the model plant *Arabidopsis thaliana*. Highest homology was noticed with transcripts from *Fragaria vesca* (39.31%), followed by *Citrus sinensis* (38.90%), *Populus trichocarpa* (38.73%), *Ricinus communis* (38.25%) and *Vitis vinifera* (38.10%) (Supplementary Table S2). A total of 68,988 (57.51%) transcripts, including their possible splice variants, showed no homology (at an *E*-value of 10^−5^) with any of the eight species, NR and Swiss-Prot databases examined in the study. These transcripts could be specific to *Litchi chinensis*.

### Functional categorization

The assembled data was classified into 25 groups by using the COG tool (Supplementary Fig. S1). This groups the sequences on the basis of evolutionary relationship among bacteria, algae and eukaryotes and provides a useful platform for protein classification of unsequenced organisms^20^. The analysis suggested that ovule development in litchi involved transcriptional, post transcriptional and metabolism related regulation. Further analysis was done by Gene Ontology (GO) tool that classifies genes by their molecular functions, biological processes in which they are involved, and the cellular location of the gene products. The 50,748 unique transcripts, showing best hits (*E*-value ≤ 10^−5^) in the aforementioned public databases, were examined with the GO tool. The transcripts, were categorized in 6,333 GO terms into biological processes, cellular components and molecular functions (Supplementary Fig. S2). In biological process category, the major representation was for metabolic processes (82.18%), cellular process (65.99%) and cellular metabolic processes (53.87%). Catalytic activity (69.30%) represented the most abundant sub-category in molecular function.

### Differential gene expression in developing ovules between bold- and small-seeded litchi genotypes

**Figure 1:**
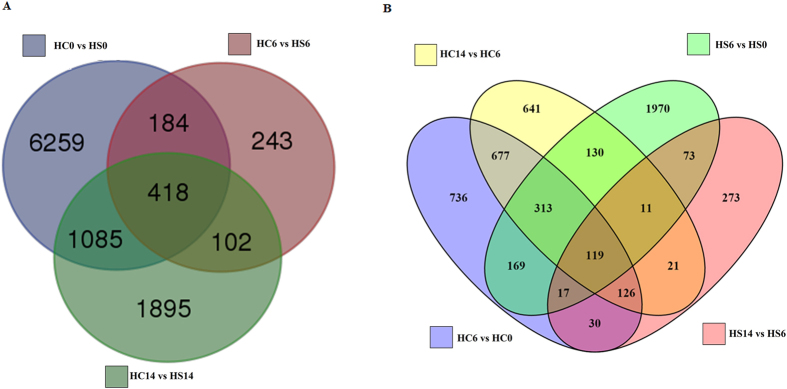
Venn diagram showing total number of the differentially expressed transcripts (log_2_ fold ≥ 2; *P*-value ≤ 0.001) between bold- (HC) and small- seeded (HS) litchi ovules at (**A**) 0, 6, and 14 DAA and (**B**) transition from 0 to 6 and 6 to 14 DAA.

The expression patterns of transcripts between the early-stage ovules of bold (HC)- and small (HS)-seeded genotypes were investigated. The highest number of differentially expressed transcripts was noticed at 0DAA. A total of 7,946 transcripts expressed differentially (log_2_ fold change ≥ 2; *P* value ≤ 0.001) at 0DAA. Pair wise comparison of the developing ovules revealed common and exclusive differentially expressed transcripts at 0, 6 and 14 DAA between the two genotypes (Fig. 1A) (Supplementary Data 1 and 2). The 6,259 differentially expressed transcripts were exclusive to 0 DAA (Fig. 1A), suggesting a dominant role of maternal factors in the regulation of seed size in litchi. The maternal genes determine the development of seed coat exclusively, while also function in co-ordination with paternal genes in the development of endosperm and embryo. The role of maternal tissues in determining seed size has been emphasized earlier^5^,^6^,^21^.

### Putative hormone related genes

**Figure 2:**
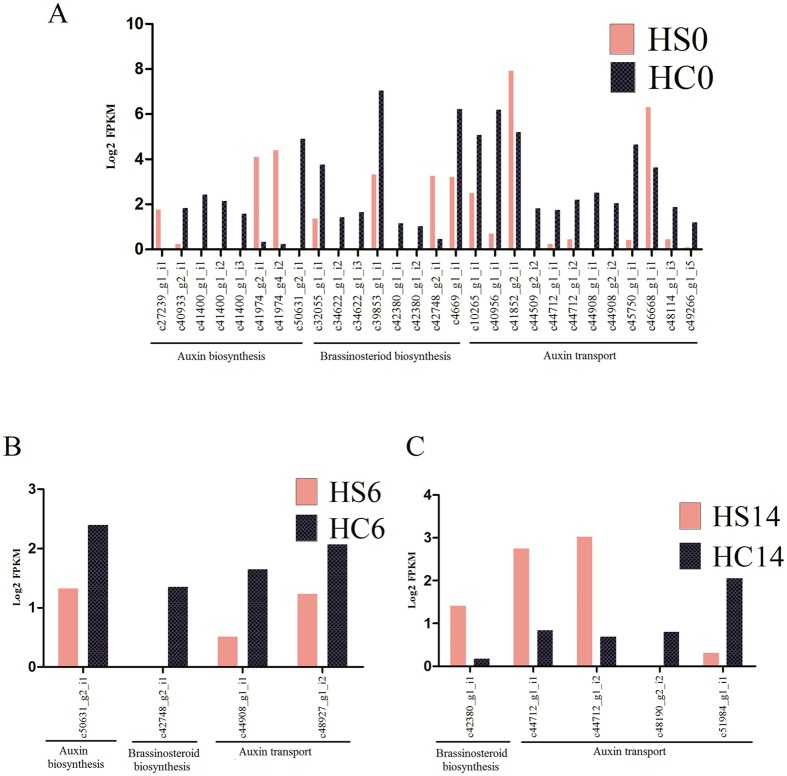
Differentially expressed (log_2_ fold ≥ 2; *P*-value ≤ 0.001) hormone-related genes at early ovule developmental stages of bold-seeded (HC) vs small-seeded (HS) litchi genotypes. Log_2_ (FPKM + 1) of putative auxin, brassinosteroid biosynthesis and auxin transport transcripts at (**A**) 0 DAA, (**B**) 6 DAA and (**C**) 14 DAA are shown.

The endogenous levels of different plant hormones such as auxins, gibberellins, cytokinins, abscisic acid, ethylene, and brassinosteroids play important roles in seed and fruit development. The BLASTx searches revealed the expression of a total of 2,331 trinity transcripts showing homology with hormone related genes in the developing ovules. The maximum representation was of the transcripts related to abscisic acid pathway followed by auxin in the ovule transcriptome data. A total of 430, 50 and 137 transcripts were differentially expressed (HC vs HS) at 0, 6 and 14 DAA, respectively. The transcripts related to auxin and brassinosteroid biosynthesis were down-regulated in small-seeded genotype at 0, 6 and14 DAA (Fig. 2A–C, Supplementary Data 3). The biosynthesis and transportation of auxin play important roles in cell division, differentiation, expansion, and determining apical to basal patterning of gynoecium^22^,^23^. Auxin accumulates at the tip of primordia from where it is transported to sub-epidermal layers through vascular route^23^. Majority of auxin transport related transcripts were down regulated at 0 and 6 DAA in the small-seeded genotype (Fig. 2A,B). Down-regulation of auxin transport in embryo sac may affect megagametogenesis and embryo patterning^24^. The putative transcripts for auxin efflux carrier family proteins such as PIN1 (AT1G73590; c40956_g1_i1&c45750_g1_i1), PIN6 (AT1G77110; c44908_g1_i1) and EIR1 (AT5G57090; c44908_g2_i2), involved in the maintenance of auxin gradient in embryo sac^24^, were down-regulated in the developing ovules of small-seeded litchi (Fig. 2A). The auxin transporter LAX2 (AT2G21050; c42412_g1_i1) regulates vascular development^25^. It was repressed in small-seeded ovules (Supplementary Table S3). Down-regulation of auxin transport in ovule may hamper megagametogenesis and embryo patterning^24^, and sometimes may induce parthenocarpy^26^. The suppression of gene expression for auxin transport could hamper proper seed development in litchi. Although we could not detect any transcript homologous to cytokinin biosynthesis pathway genes, in developing ovule of small-seeded litchi at 0 DAA, the candidate genes for cytokinin transport were up regulated. The higher level of cytokinin transport could make ovular tissues more sensitive to various stresses^27^. Reproductive tissues of flowering plants are sensitive to different stresses, which may influence ovule development^28^. Several transcripts which showed no expression in the ovule of bold-seeded genotype were significantly expressed in small-seeded litchi and were homologous to stress related genes at 0 DAA (Supplementary Table S4). This suggests that the small-seeded ovule experiences a metabolic state of stress, which subsequently hampers seed development.

Brassinosteroids play important role in initiation and formation of reproductive organs. These regulate stress tolerance, stomatal development and vascular differentiation^29^,^30^,^31^,^32^. Their role has been discussed in determining seed size by regulating endosperm growth and maternal imprinting^33^. Sterol methyltransferase 2 (c4669_g1_i1_AT1G20330) is involved in sterol biosynthesis and the loss of function leads to defective cotyledon growth^34^. DWARF1 (AT3G19820; c39853_g1_i1) is involved in the conversion of brassinosteroid precursor 24-methylenecholesterol to campesterol, and the loss of function induces dwarf phenotype, sterility and seed abortion^35^. We found relatively repressed expression of the putative genes for DWARF1 and sterol methyltransferase 2 in small-seeded litchi at 0DAA (Fig. 2A). The results agree with the up- and down-regulation of transcripts at 0 DAA for candidate genes identified as negative (IKU1_AT2G35230; c50429_g1_i1) and positive (KLU_ AT1G13710; c46160_g1_i3 & c46160_g1_i1 and ANT_AT4G37750; c46773_g2_i3) regulators of seed size in *Arabidopsis*^5^,^36^ (Supplementary Table S3). Putative transcript for brassinosteriod deactivation, sulphotransferase 12^37^ expressed at higher level at 14 DAA in the small-seeded genotype.

### Putative transcription factor genes

Transcription factors (TFs) are key regulators of spatial, temporal and quantitative gene expression. The BLASTx analysis revealed a total of 2,155 trinity transcripts showing homology with TFs involved in early-stage ovule development in bold- and small-seeded litchi. The MYB family TFs were most abundant in the ovule transcriptome data of litchi. A total of 426, 33 and 125 TF related transcripts were differentially expressed (bold seeded vs small seeded) at 0, 6 and 14 DAA, respectively.

**Figure 3:**
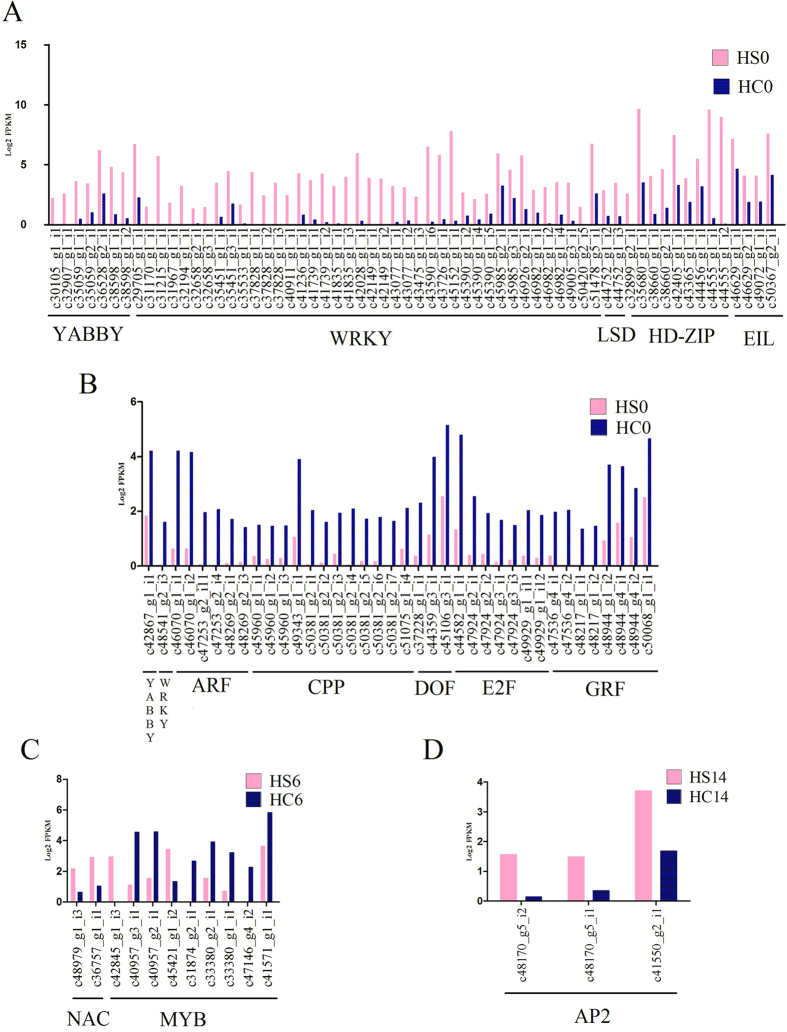
Differentially expressed (log_2_ fold ≥ 2; *P*-value ≤ 0.001) putative transcription factor genes in early ovule developmental stages of bold-seeded (HC) vs small-seeded (HS) litchi genotypes. Log_2_ (FPKM + 1) of the genes (**A**) upregulated in small-seeded litchi genotype at 0DAA; (**B**) down-regulated in small-seeded litchi genotype at 0 DAA; (**C**) NAC and MYB transcription factors at 6 DAA and (**D**) AP2 family transcription factors at 14 DAA.

The expression of members of YABBY, WRKY, LSD, HD-ZIP and EIL family TFs was up-regulated in small-seeded litchi at 0 DAA (Fig. 3A, Supplementary Data 4). The YABBY family regulates polar differentiation of abaxial side of lateral organs, playing important role in organ morphogenesis^38^,^39^. Out of six YABBY genes known in *Arabidopsis* genome, four (FIL, YAB2, YAB3 and YAB5) are expressed in leaf and leaf derived organs, and two (INO and CRC) are restricted to floral organs^39^. The putative genes for FIL (c35059_g1_i1, c35059_g2_i1, c38598_g1_i1&c38598_g1_i2; AT2G45190), YAB2 (c36528_g2_i1_AT1G08465), YAB5 (c32907_g1_i1_AT2G26580), and CRC (c30105_g1_i1_AT1G69180) showed higher expression in the ovules of small-seeded genotype (Fig. 3A). CRC is expressed in placental tissue for proper carpel identity and nectar development^39^,^40^. In contrast, putative INO (c42867_g1_i1_AT1G23420) was down-regulated in the ovules of small-seeded genotypes (Fig. 3B). INO plays important role in the development of outer integuments, and its down-regulation leads to seedless phenotype in *Arabidopsis*^41^ and *Annona squamosa*^42^.

WRKY family of TF are involved in various stress responses and plant developmental processes^43^. Putative transcripts of LSD one like 1 gene (c44752_g1_i2 &c44752_g1_i3; AT1G32540) was up-regulated in small-seeded genotype (Fig. 3A). LSD one like 1 is known to cause stress induced cell death in *Arabidopsis thaliana*^44^. HD-ZIP is involved in various developmental processes in plants^45^. HB-5, a member of HD-ZIP (c35680_g1_i1_AT4G36740), induced during ovule abortion^46^, was highly expressed in small-seeded genotype. EIL family members regulate various genes involved in developmental processes and stress response^47^. Putative transcripts for ethylene-insensitive3-like3 (c46629_g2_i1&c46629_g1_i1; AT1G73730) and ethylene insensitive 3 (c49072_g1_i1 & c50367_g2_i1; AT3G20770) involved in ethylene signaling were up-regulated in small-seeded genotype (Fig. 3A). In *Arabidopsis thaliana*, ethylene insensitive 3 is potent to activate ethylene pathway in absence of ethylene^48^. Ethylene responsive genes are found to be up-regulated in stress induced ovule abortion^47^.

The TF families, ARF, CPP, Dof, E2F and GRF, were down regulated in small-seeded genotype at 0DAA (Fig. 3B, Supplementary Data 4). ARFs activate or repress genes in response to auxins^49^ CPP family plays important role in regulating development of reproductive tissues and cell division^50^. TSO 1 (c45960_g1_i1, c45960_g1_i2, c45960_g1_i3, c49343_g1_i1, c50381_g2_i1 & c50381_g2_i4; AT3G22780), TSO1-like CXC2 (c50381_g2_i2, c50381_g2_i3, c50381_g2_i5, c50381_g2_i6 & c51075_g1_i4; AT4G14770) and TSO1-like CXC domain containing proteins (c50381_g2_i7_AT3G04850) of CPP family were down regulated in small-seeded genotype (Fig. 3B). TSO 1 and TSO1-like CXC are essential for development of reproductive tissue and cell cycle control^51^,^52^. E2F proteins regulate transcription of genes expressed in a quiescent stage^53^. Putative transcripts of E2F transcription factor family E2F3 (c47924_g2_i1&c47924_g2_i2; AT2G36010), E2F-like2 (c49929_g1_i11& c49929_g1_i12; AT3G01330), E2F-like3 (c44582_g1_i1_ AT3G48160) and E2F1 (c47924_g3_i1&c47924_g3_i3; AT5G22220) are key components of cell cycle regulation^54^. These were down-regulated in small-seeded genotype (Fig. 3B). GRF family members regulate cell expansion and its mutants develop smaller cotyledon size^55^. The results indicate up-regulation of several stress related TFs (Fig. 3A) in the ovules of small-seeded genotype.

**Figure 4:**
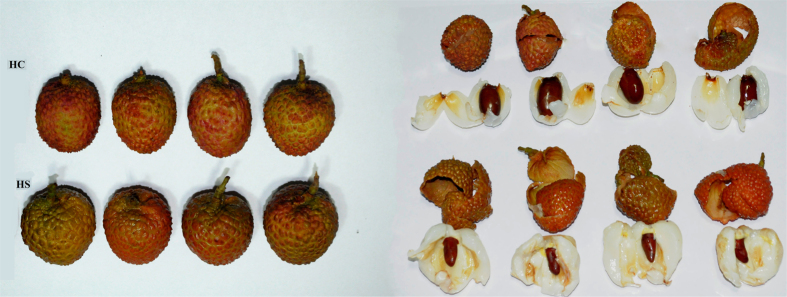
Comparison of seed size and fruit parts in mature fruits in the bold - and small -seeded litchi genotypes at 60 to 65 DAA. Fruits of small-seeded (HS) litchi show thicker pulp in fruits, as compared to the bold-seeded genotype (HC).

The candidate genes for NAC TFs were up-regulated in small-seeded litchi at 6 DAA (Fig. 3C, Supplementary Data 4). NAC transcription factors participate in the regulation of seed development and stress responses^56^,^57^. The putative transcripts for MYB family of TF were down-regulated in small-seeded genotype (Fig. 3C). MYB transcription factors regulate biosynthesis of flavonoids in grapes, required for proper pollination and embryo development in plants^58^. The A-class homeotic gene APETALA2 (AP2), a floral homeotic TF, is known to negatively regulate seed size but promote integument growth^59^. AP2 was up-regulated in small-seeded genotype at 14 DAA (Fig. 3D). This is in agreement with the development of fruits with under developed seed and well-developed arils of integument origin (Fig. 4), in the small seeded litchi.

### Ovule identity determining genes

Based on homology with the genes that have been attributed role in ovule development and embryogenesis in *Arabidopsis thaliana*, we mapped the transcripts, expressed differentially between the bold- and small seeded genotypes, on a model ovule developmental pathway. Higher expression of NGATHA (AT2G46870; c41974_g2_i1 & c41974_g4_i2), BEL1-like (AT2G35940; c45509_g1_i1, c45509_g1_i2, c45509_g1_i3, c39557_g3_i1 & c39557_g3_i2), HB-5 like (AT4G36740; c35680_g1_i1), LOB domain containing protein (AT2G28500; c36314_g1_i1) and HB-7 like gene (AT2G46680; c44555_g1_i1 & c44555_g1_i2) was noticed in the small-seeded litchi at 0DAA (Fig. 2A, Supplementary Table S3). BEL1-like homeodomain 1 protein controls ovule patterning through auxin signaling^60^. Its ectopic expression in embryo sac leads to defects in nuclear migration, cellularization and embryogenesis^61^. Ectopic expression of LOB domain containing proteins in flowering tissues causes alteration of flower size and shape and may lead to seed abortion^62^. The *Arabidopsis* mutants, lacking differentiated gametophyte, exhibit higher level of expression of HB-7^63^. NGATHA regulates auxin-mediated development of female reproductive structures in plants. Its overexpression has been reported to reduce organ growth e.g. leaves, flowers and cotyledons^64^.

Cell cycle is tightly co-regulated with cell patterning, and morphogenesis^65^. The candidate genes involved in the cell cycle regulation, cyclin D1 (AT1G70210; c47767_g1_i1 & c47767_g3_i1), cyclin A3 (AT1G47230; c46681_g1_i1) cyclin D4 (AT5G65420; c47767_g1_i2), dUTP pyrophosphatase (AT3G46940; c26922_g1_i1 & c38696_g1_i1), E2F target gene 1 (AT2G40550;c46496_g1_i1), minichromosome maintenance chromosome 5 (MCM5) (AT2G07690; c46324_g1_i1), ribonucleoside-diphosphatereductase small chain C/TSO2 (AT3G27060; c39450_g1_i1), DNA primase/POLA3 (AT5G41880; c41508_g1_i1,c41508_g1_i2 & c41508_g2_i1) and RPA70B (AT5G08020; c37107_g1_i1) were down-regulated in litchi small-seeded genotype at 0 DAA (Supplementary Table S3). E2F target gene 1 controls cell cycle progression by cohesion of sister chromatid and DNA repair^66^. Minichromosome maintenance 5 is involved in initiation of DNA replication in cell^67^. No reads were detected for fructose-bisphosphatealdolase 2 (AT4G38970; c43203_g3_i1), required for biomass accumulation^68^ in the developing ovules (0, 6, and 14 DAA) of small-seeded genotype. At hormone level, the events of cell division, differentiation and expansion in plant tissues are regulated by auxin^23^. The transcriptional pattern in the developing ovules of small seeded genotypes showed suppression of auxin transport.

### Embryogenesis related genes

In litchi, the preliminary zygotic cell division happens during 3 to 7 DAA, and the embryo enters globular stage from 14 to 21 DAA^69^. We investigated expression pattern of several embryogenesis related genes in the two contrasting litchi genotypes. Ole e 1 allergen and extensin family protein (AT2G34700; c38654_g1_i1), involved in pollen tube guidance during fertilization^70^, was down-regulated in developing ovules of the small-seeded litchi (Supplementary Table S3). The flavin binding monooxygenase (AT5G25620; c50631_g2_i1 & c41400_g1_i3) that provides ROS tolerance^71^ during pollination was down regulated in the small seeded genotype (Fig. 2A). Asymmetric cell division of zygote is very important for embryo development and auxin plays an important role in the establishment of polarity^72^. Expression of Topless/TPR2 (AT3G16830; c51185_g3_i1, c51185_g3_i2, c51185_g3_i3 and c51185_g3_i4) was suppressed in small-seeded litchi genotype (Supplementary Table S3). In the loss of function mutant of this gene, axis establishment is severely affected in *Arabidopsis*^73^. The putative gene was down–regulated in the small-seeded genotype. EP3 chitinase (AT3G54420; c45503_g1_i1 & c45503_g1_i2) and phytosulfokine 2/PSK2 (AT2G22860; c79596_g1_i1) hamper embryo development by inducing somatic embryogenesis^74^. These genes showed higher expression in small-seeded litchi genotype at 0 DAA (Supplementary Table S3).

**Figure 5:**
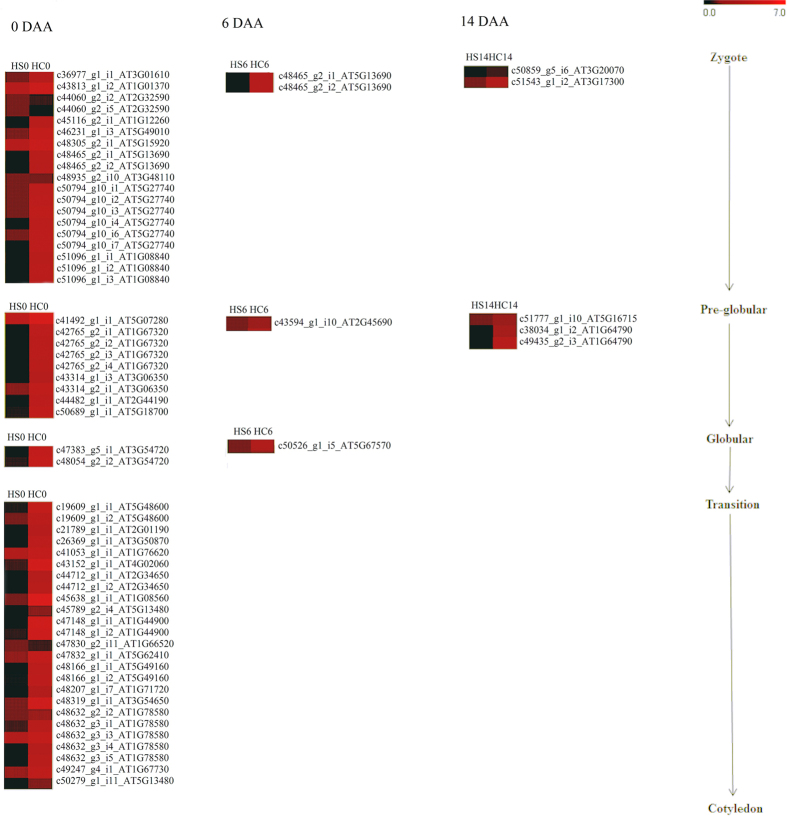
Functional mapping of transcripts that express differentially between the small (HS) and bold (HC) seeded litchi genotypes. The function has been assigned based on the phenotypes reported in *Arabidopsis thaliana* following loss of function mutations in homologous genes. Heatmaps in different columns represent the expression levels in ovule at 0, 6 and 14 DAA.

Embryogenesis begins with the fertilization of egg, forming zygote. It undergoes asymmetrical division, followed by defined pattern of embryo growth to subsequently form cotyledon^75^. Essential genes for seed development have been identified in *Arabidopsis thaliana* by analyzing the mutants that severely affect embryo developmental stages^76^. A total of 440 seed development related *Arabidopsis* – like transcripts were identified in the litchi transcriptome data. Such litchi transcripts, that appear to effect embryo development at pre-globular (AT3G01610, AT1G01370, AT2G32950, AT1G12260, AT5G49010, AT5G15920, AT5G13690, AT5G27740 and, AT1G08840), globular (AT5G07280, AT1G67320, AT3G06350, AT2G44190 andAT5G1870), transition (AT3G54720) and cotyledon stage (AT5G48600, AT2G01190, AT3G50870, AT4G02060, AT2G34650, AT1G08560, AT5G13480, AT1G44900, AT1G76620, AT1G66520, AT5G62410, AT5G49160, AT1G71720, AT3G54650, AT1G78580 and AT1G67730) were down-regulated at 0DAA in small-seeded litchi genotype (Fig. 5, Supplementary Table S5). Replication factor C subunit 3 (AT5G27740; c50794_g10_i1, c50794_g10_i2, c50794_g10_i3, c50794_g10_i4 and c50794_g10_i6) is a clamp loader during replication. Its mutagenesis causes severe defects in embryo development at pre-globular stage in *Arabidopsis thaliana*^77^. COP 1 (AT2G32950; c44060_g2_i2 & c44060_g2_i5) regulates seed development by suppressing expression of photomorphogenic pathway and enhancing expression of dark induced seed development pathway^78^. N-acetyl–glucosaminidase catalyzes the breakdown of sugars in proteoglycans, and mutation in the gene may induce embryo defects at pre-globular stage^79^. N-acetyl–glucosaminidase/CYL 1 (AT5G13690; c48465_g2_i1 & c48465_g2_i2) was down-regulated at 0 and 6 DAA in small-seeded genotype. Histone H3 variant (AT1G01370; c43813_g1_i1) is important for kinetochore assembly and chromosome segregation, its knock out causes small seed with reduced fertility^80^. Structural maintenance of chromosome 5 (AT5G15920; c48305_g2_i1) is involved in DNA double strand repair^81^. Endosperm defective 1 (AT2G44190; c44482_g1_i1) is a microtubule-associated protein, essential for cell division of embryo and endosperm^82^. RUNKEL (AT5G18700; c50689_g1_i1) is responsible for phragmoplast organization and cell plate expansion^83^. ALTERED MERISTEM PROGRAM 1 (AT3G54720; c47383_g5_i1 & c48054_g2_i2) regulates pollen tube attraction by synergid cells^84^. Structural maintenance of chromosomes protein 4 (AT5G48600; c19609_g1_i1 & c19609_g1_i2) plays important role in condensation of chromatin during mitosis^85^. Trehalose-6-phosphate synthase 1 (AT1G78580; c48632_g2_i1, c48632_g2_i2, c48632_g2_i3, c48632_g2_i4 & c48632_g2_i5) regulates trehalose synthesis, and is essential for seed maturation^86^. MMC 2 (AT1G44900; c47148_g1_i1 & c47148_g1_i2) is a cell cycle regulator that plays important role in stem cell maintenance and differentiation^87^. F BOX-LIKE 17 (AT3G54650; c48319_g1_i1) positively regulates cell proliferation^88^. At 6 DAA, transcripts causing embryo defects leading to terminal phenotypes at pre-globular (AT5G13690; c48465_g2_i1 & c48465_g2_i2), globular (AT2G45690; c43594_g1_i10) and transition stage (AT5G67570; c50526_g1_i5) were down-regulated in the small-seeded litchi cultivar (Fig. 5). Mutation in the gene encoding Shrunken Seed protein (AT2G45690), which is involved in peroxisome assembly and protein trafficking, leads to shrunken seeds^89^. Pentatricopeptide repeat containing protein, delayed greening 1 (AT5G67570; c50526_g1_i5) is a regulator of early chloroplast development and gene expression^90^. The homologues of transcripts causing embryo development at pre-globular (AT3G20070; c50859_g5_i6 and AT3G17300; c51543_g1_i2) and globular stage (AT5G16715; c51777_g1_i10 and AT1G64790; c38034_g1_i2 & c49435_g2_i3) were down-regulated in the small-seeded litchi genotype at 14 DAA (Fig. 5). However, the master regulatory genes, if any, that may lead to differential expression of the genes downstream have not yet been identified. Several genes that expressed differentially between HC and HS could not be functionally annotated due to lack of homology with the database. A total of 3,413 differentially expressed transcripts could not match with any known protein in the database. These unknown transcripts could be important for the development of seeds in litchi.

**Figure 6:**
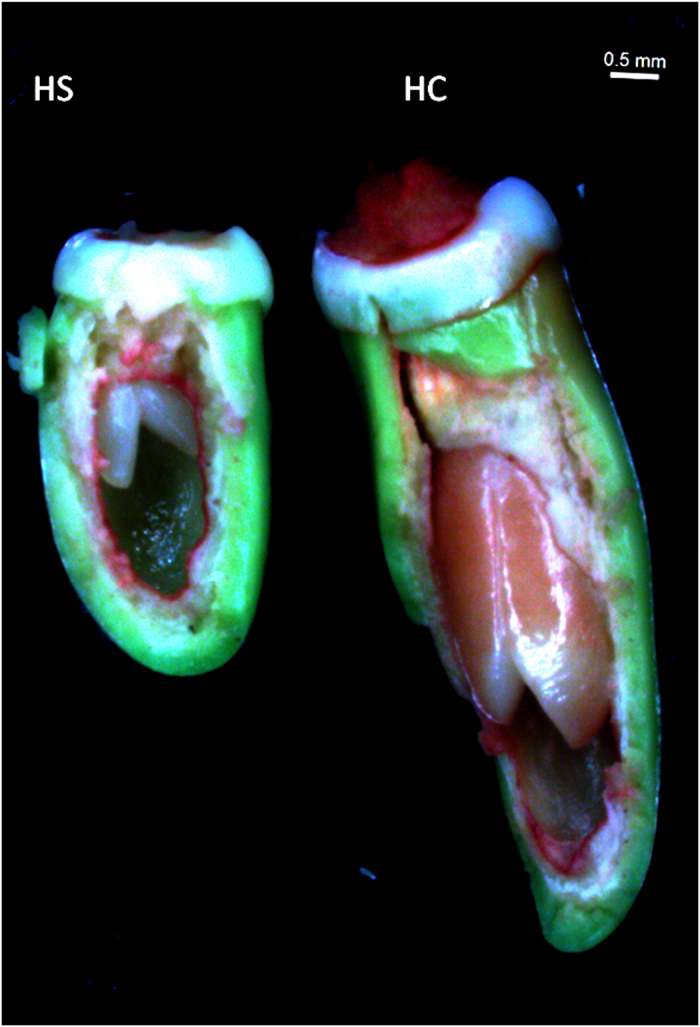
Under- and normally-developed embryos in small- (HS) and bold-seeded (HC) genotypes in ovules at 35 DAA.

A distinct expression pattern of the genes in early-stage ovule development might lead to under-developed embryos (Fig. 6), resulting into the development of small seed size in litchi (Fig. 4).

### Shift in gene expression during ovule development

As shown in Fig. 1B, during transition from 0 to 6 DAA, a total of 618 differentially expressed transcripts were common between bold and small-seeded ovules; whereas, 1,569 and 2,184 differentially expressed transcripts were exclusively present in the ovules of bold- and small-seeded genotypes, respectively. During transition from 6 to 14 DAA a total of 277 differentially expressed transcripts were common between bold and small seeded ovules; whereas, 1,761 and 393 differentially expressed transcripts were exclusive to bold and small-seeded ovules, respectively (Fig. 1B). In developing ovules, zygotic division is a metabolically active process at which apical-basal body of the polarized zygote is established by asymmetric divisions. The Homeobox proteins, WRKY and receptor kinases are known to express at high level during zygotic division^91^,^92^,^93^. The homologues of these genes were up-regulated in large-seeded genotype at 6 DAA as compared to 0 DAA ovules, in contrast to the small-seeded ovules, where these were down regulated. Expression of auxin, gibberellic acids and brassinosteriods was up-regulated at 6 DAA and then decreased at 14 DAA in bold-seeded genotype, which is in accordance with the pattern of expression in an earlier report on litchi^94^. No such trend was observed in small-seeded genotype, with little change in expression pattern between 6 to 14 DAA (Supplementary Fig. S3).

The expression differences between the developmental stages of ovules may lead to partial or complete abortion of seed in litchi.

### Validation of ovule-specific differentially expressed genes obtained from RNA-seq libraries in two genotypes with contrasting seed size

**Figure 7:**
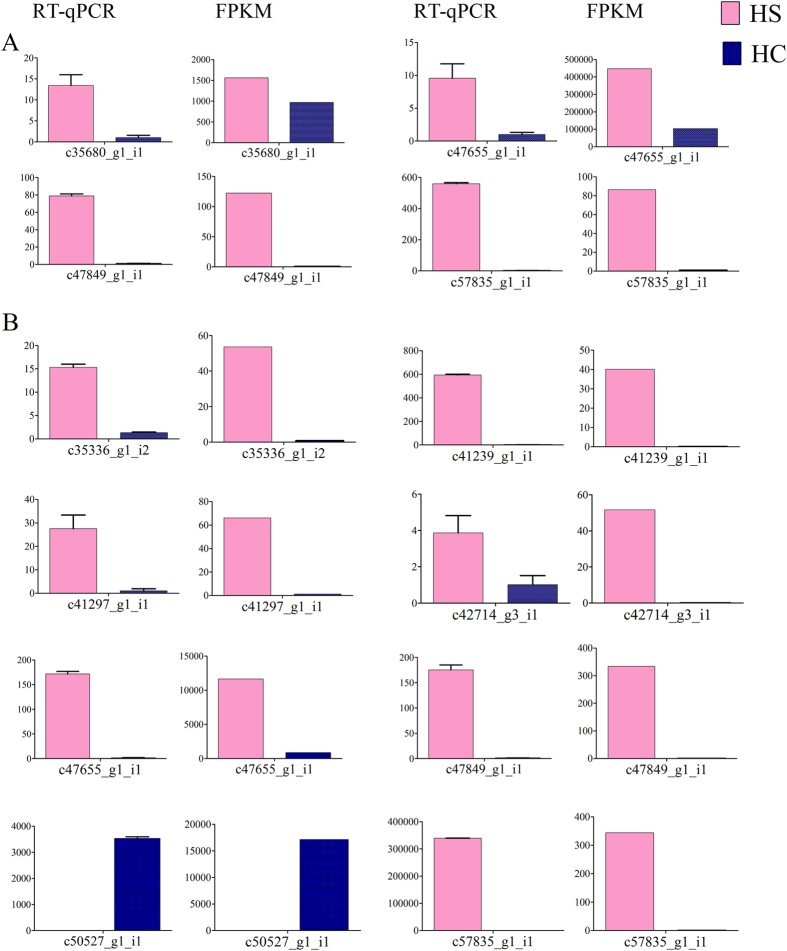
RT-qPCR validation of some genes expressed differentially during ovule development between HC and HS. The RT-qPCR (left) and FPKM (right) values of (**A**) four differentially expressed genes at 6 DAA and (**B**) eight differentially expressed genes at 14 DAA are compared.

To validate the differential expression profiles identified in the digital data obtained by RNA-seq, we performed in real time PCR on 4 representative transcripts selected for different levels of differential expression at 6, 8 at 14 DAA (Fig. 7). The representative transcripts were selected from among both annotated and non-annotated entries and with contrasting levels of expression in the two genotypes (Supplementary Table S6). The expression pattern obtained in real time assay was comparable with that analyzed from RNA-seq data. The high and significant Pearson correlation coefficient (0.808) of RNA-seq with real time results suggests high confidence in the expression values obtained from the transcriptome data. Hence, the RNA-seq data can be used reliably for relative expression analysis of genes in seed development pathway in litchi.

In conclusion, we report the first transcriptome information on early-stage ovule development in litchi genotypes with contrasting seed size. We identified various differentially expressed genes that showed homology to the genes reported in database. Our analysis shows that the genes involved in endosperm development as well as embryo development were associated with small seed size in the small-seeded genotype of litchi. Such genes were related to hormone biosynthesis pathways, transcription factors, ovule identity determinants, and embryogenesis. The developing ovules of small seeded genotype showed higher expression of several stress related transcripts, suggesting the association of metabolic stress with improper seed development in litchi. A number of genes expressed differentially at 0 DAA, suggesting that some factors resident in maternal tissue may set in the process that leads to the reduced seed size in the small-seeded litchi genotypes.

## Materials and Methods

### Plant materials and sample collection

Plant materials were collected from Fruit Research Station, Punjab Agriculture University, Hoshiarpur, Punjab, India. Two litchi genotypes of contrasting seed size - Calcuttia (bold seeded) and Seedless Late (small seeded), were selected for the study. In our previous study, these two genotypes were reported to be closely genomically related, with the Nei 72 genetic distance of 0.36^95^. The hermaphrodite female flowers, with two lobed styles and sticky stigma, were identified on the panicle as highly receptive flower stage and marked as 0 DAA (days after anthesis). Developing fruits at 0, 6, and 14 DAA were harvested randomly from three healthy trees each, of the two genotypes. The samples were surface sterilized with absolute ethanol before harvesting, as described previously^7^. The harvested samples were immediately frozen in liquid nitrogen and stored at −80 °C until RNA extraction.

### RNA isolation and library construction for transcriptome analysis

Ovules were excised from 20–30 developing fruits at each developmental stage, in chilled (−20 °C) ethanol, under Leica M205 C stereo microscope in RNase free condition (Supplementary Figs S4 and S5). Total RNA was extracted by using Spectrum Plant Total RNA kit (Sigma Aldrich, St. Louis, MO, USA), following manufacture user’s protocol. DNase I (Sigma Aldrich St. Louis, MO, USA) treatment was given to remove genomic DNA contamination. The RNA integrity was established, using a 2100 Bioanalyzer (Agilent Technologies, Santa Clara, CA, USA). The equivalent quantity of total RNA extracted from the ovules of three biological replicates (plants) was pooled for transcriptome sequencing.

### Transcriptome sequencing and *de novo* assembly

Paired-end Illumina mRNA libraries were generated from total RNA samples from ovules at 0, 6, and 14 DAA for the two genotypes in accordance with the manufacturer’s instructions (Illumina Inc., USA). Transcriptome sequencing was performed on IlluminaHiSeq2000 platform (Sandor, Hyderabad, India). The raw reads were pre-processed by removing adaptor sequences, and discarding empty reads and low-quality sequences. The high quality paired end 101 bp reads of each sample were then used for transcriptome *de novo* assembly using Trinity pipeline (release 2015-02-14) at default parameters. The sequences assembled by Trinity (Trinity transcripts) are referred to as transcripts in the manuscript.

### Comparative analysis

A comparative analysis was performed by using the litchi transcripts as queries (BLASTx; *E* value ≤ 10^−5^) against the protein databases of crops such as, *Arabidopsis thaliana* (ftp://ftp.psb.ugent.be/pub/plaza/plaza_ public_dicots_ 03/Fasta/proteome. ath.tfa.gz), *Citrus sinensis* (ftp://ftp.psb.ugent.be/pub/plaza/plaza_public_dicots_03/Fasta/proteome.csi. tfa.gz). *Ricinus communis* (ftp://ftp.psb. ugent. be/pub/plaza/plaza_public_ dicots_03/Fasta/proteome. rco.tfa.gz), *Populus trichocarpa* (ftp://ftp.psb. ugent.be/pub/plaza/plaza_public_ dicots_03/Fasta/proteome.ptr.tfa.gz), *Fragaria vesca* (p.t. be/pub/plaza/plaza_public_ dicots_03/Fasta/proteome. fve.tfa.gz), *Carica papaya* (ftp://ftp.psb.ugent.be/pub/plaza/plaza_ public_dicots_03/Fasta/proteome.cpa.tfa.gz) and *Glycine max* (t.a_public_dicots_03/Fasta/proteome.gma. tfa.gz).

### Functional annotation

Functional annotation of the transcripts was performed by BLASTx (*E*-value ≤ 10^−5^) against databases: NCBI non-redundant (nr) database (http://www.ncbi.nlm.nih.gov), Swiss-Prot protein database (http://www.expasy.ch/ sprot), and Clusters of Orthologous Groups of proteins (http://www.ncbi. nlm.nih.gov/COG). The process was accelerated using WImpiBLAST tool^96^ on institute’s high performance computing cluster.

The litchi transcripts showing homology with the protein database of different crops (*Arabidopsis thaliana, Citrus sinensis, Populus trichocarpa, Ricinus communis, Glycine max, Fragaria vesca, Carica papaya and Vitis vinifera*) were used for GO term assignment.

### Gene ontology (GO) analysis

GO terms for the litchi transcripts were obtained from PLAZA (http://bioinformatics.psb.ugent.be/plaza/versions/plaza_v3_dicots/download/index), using the corresponding IDs of aforementioned eight crops. The GO terms were used as inputs in the agriGO analysis tool (http://bioinfo.cau.edu.cn/agriGO/analysis.php)^97^ for GO categorization (P value ≤ 0.05) with customized annotation option. The GO (P value ≤ 0.05) was given as input in the REVIGO tool (http://revigo.irb.hr/)^98^, results of REVIGO were obtained in.csv format and used for histogram preparation.

### Transcript, abundance estimation and identification of differentially expressed transcripts

The *de novo* assembled transcripts were used as a reference to map the individual Illumina reads, using script ‘align_and_estimate_abundance.pl’ bundled with Trinity suite. Abundance estimation was done with the RSEM version 1.2.9^99^, at default parameter using BOWTIE2 version 2.1.0^100^ mapping. Relative measure of transcript abundance was obtained as TPM (Transcripts per million) and FPKM (Fragments per Kilobase of transcripts per Million mapped reads). Expression levels of different transcripts were compared among the samples by edgeRBioconductor^101^, using script run_DE_analysis.pl at default parameter. EdgeR performs an additional TMM (Trimmed Mean of M-Values) scaling normalization to examine differences in total RNA production across the samples^102^.

EdgeR Bioconductor uses negative binomial distribution as statistical model for identification of possible differentially expressed transcripts in the absence of biological replicates^103^.

### Quantitative real-time PCR analysis

Differentially expressed transcripts obtained by high throughput sequencing were validated by quantitative real-time PCR. Total RNA were isolated from ovules at 6 and 14 DAA, and cDNA was synthesized from total RNA (500 ng) using oligo (dT) primers and M-MLV reverse transcriptase, according to the manufacturer’s instructions (Invitrogen, USA) in a 20 μl volume. Between the two libraries (HC and HS), four and eight differentially expressed genes at 6 DAA and 14 DAA respectively, were selected to observe their expression patterns between the two cultivars (Supplementary Data 5). Transcript levels were analyzed by quantitative real-time PCR using the fast SYBR green master mix (Applied Biosystems, USA) and an ABI 7500 Real-Time PCR System (Applied Biosystems, USA) according to the manufacturers’ instructions. All biological replicates were analyzed in duplicate. Real-time PCR reactions were normalized to the Ct values for litchi LcActin (HQ615689). The relative expression levels of the target genes were calculated using the formula 2^−ΔΔCT^.

### Data Availability

Raw sequencing data is available through NCBI Sequence Read Archive, accession number: SRP076141. All samples were sequenced as 101-nucleotide paired-end reads on an Illumina HiSeq2000 sequencer.

## Additional Information

**How to cite this article**: Pathak, A. K. *et al*. Transcriptional changes during ovule development in two genotypes of litchi (*Litchi chinensis* Sonn.) with contrast in seed size. *Sci. Rep*. **6**, 36304; doi: 10.1038/srep36304 (2016).

**Publisher’s note:** Springer Nature remains neutral with regard to jurisdictional claims in published maps and institutional affiliations.

## Supplementary Material

Supplementary Information

Supplementary Data 1

Supplementary Data 2

Supplementary Data 3

Supplementary Data 4

Supplementary Data 5
