# Supplementary material for: Transcriptional changes during ovule development in two genotypes of litchi (Litchi chinensis Sonn.) with contrast in seed size
Source: Sci Rep. 2016 Nov 8;6:36304. doi: 10.1038/srep36304 (PMC5099886; doi:10.1038/srep36304)
Supplement: Supplementary Information [file srep36304-s1.pdf]

Transcriptional changes during ovule development in two genotypes of litchi (*Litchi chinensis* Sonn.) with contrast in seed size

Ashish K. Pathak[1,4], Sudhir P. Singh[1,3], Yogesh Gupta[1], Anoop K.S. Gurjar[1], Shrikant S. Mantri[1], Rakesh Tuli[1,2*]

[1]National Agri-Food Biotechnology Institute (NABI), Department of Biotechnology (DBT), Mohali, Punjab, India

[2] University Institute of Engineering and Technology, Panjab University, Chandigarh, India

[3]Center of Innovative and Applied Bioprocessing (CIAB), Department of Biotechnology (DBT), Mohali, India

[4]Department of Biotechnology, Panjab University, Chandigarh-160014, India

Supplementary Table S1. Summary statistics of litchi ovule specific transcriptome sequencing and assembly.

<table>
<tr><th>Sample name</th><th>Raw reads</th><th>Q 20 (%)</th><th>Trinity transcripts</th><th>Trinity genes</th><th>Average transcript</th><th>Median transcript length</th><th>GC content (%)</th><th>Total assembled reads</th></tr>
<tr><td>HS0</td><td>39,352,778</td><td>96.26</td><td rowspan="6">1,19,939</td><td rowspan="6">87,072</td><td rowspan="6">865.38</td><td rowspan="6">489</td><td rowspan="6">39.12</td><td rowspan="6">303,778,968</td></tr>
<tr><td>HS6</td><td>40,879,236</td><td>94.43</td></tr>
<tr><td>HS14</td><td>44,519,992</td><td>97.13</td></tr>
<tr><td>HC0</td><td>38,765,560</td><td>97.41</td></tr>
<tr><td>HC6</td><td>98,639,344</td><td>95.71</td></tr>
<tr><td>HC14</td><td>41,622,058</td><td>97.34</td></tr>
</table>

Supplementary Table S2. Number and percentage (in bracket) of transcripts in litchi ovule specific transcriptome from BLASTx search against eight publicly available protein databases (*Arabidopsis thaliana*, *Citrus sinensis*, *Ricinus communis*, *Populus trichocarpa*, *Glycine max*, *Fragaria vesca*, *Carica papaya* and *Vitis vinifera*) with filtering criteria of *E*-value $\leq 10^{-5}$

**Database**	**Number of transcripts annotated**
Fragaria vesca	47,150 (39.31%)
Citrus sinensis	46,662 (38.90%)
Populus trichocarpa	46,463 (38. 73%)
Ricinus communis	45,878 (38.25%)
Vitis vinifera	45,697 (38.10%)
Carica papaya	44,929 (37.45%)
Arabidopsis thaliana	44,216 (36.85%)
Glycine max	31,860 (26.56%)

Supplementary Table S3. Putative hormone, seed size, ovule identity determination, cell cycle and embryogenesis related transcripts differentially expressed between small– (HS) and large-(HC) seeded litchi genotype at 0 DAA.

**Transcript ID**	**HS0 (FPKM)**	**HC0 (FPKM)**	**Putative gene**	**Predicted function**
c42412_g1_i1_AT2G21050	0.03	26.85	LAX2	Hormones
c50429_g1_i1_AT2G35230	5.14	0.73	IKU1	Seed size
c46160_g1_i1_ AT1G13710	0.13	4.06	KLU	
c46160_g1_i3_AT1G13710	0	1.84		
c46773_g2_i3_AT4G37750	0.68	23.92	ANT	
c45509_g1_i1_AT2G35940	117.63	0.93	BEL1	Ovule identity determination
c45509_g1_i2_AT2G35940	246.85	2.03		
c45509_g1_i3_ AT2G35940	253.09	1.74		
c39557_g3_i1_ AT2G35940	52.25	0.04		
c39557_g3_i2_ AT2G35940	51.39	0.05		
c35680_g1_i1_AT4G36740	797.95	10.54	HB-5	
c36314_g1_i1_AT2G28500	59.93	0	LBD 11	
c44555_g1_i1_ AT2G46680	771.42	0.44	HB-7	
c44555_g1_i2_ AT2G46680	511.29	0.05		
c47767_g1_i1_ AT1G70210	0	8.59	Cyclin D1	Cell cycle
c47767_g3_i1_ AT1G70210	0	32.17		
c47767_g1_i2_ AT5G65420	0.13	17.18	Cyclin D4	
c46681_g1_i1_ AT1G47230	0.32	23.97	Cyclin A3	
c26922_g1_i1_ AT3G46940	0	23.22	dUTP pyrophosphatase	
c38696_g1_i1_ AT3G46940	2.64	209.11		
c46496_g1_i1_ AT2G40550	0.3	29.07	E2F target gene 1	
c46324_g1_i2_ AT2G16440	0.33	35.33	MCM5	
c39450_g1_i1_ AT3G27060	1.33	68.45	TSO2	
c41508_g1_i1_ AT5G41880	0	11.46	POLA3	
c41508_g1_i2_ AT5G41880	0	7.43		
c41508_g2_i1_ AT5G41880	0.13	16.43		
c37107_g1_i1_ AT5G08020	0.26	46.09	RPA70B	
c38654_g1_i1_ AT2G34700	3.62	175.47	Ole e 1	Embryogenesis related
c51185_g3_i1_ AT3G16830	0	4.14	TPR2	
c51185_g3_i2_ AT3G16830	0	1.06		
c51185_g3_i3_ AT3G16830	0	2.02		
c51185_g3_i4_ AT3G16830	0	1.03		
c45503_g1_i1_ AT3G54420	636.37	0.16	EP3 chitinase	
c45503_g1_i2_ AT3G54420	605.26	0.03		
c79596_g1_i1_ AT2G22860	175.16	1.01	PSK2	

Supplementary Table S4. Litchi transcripts homologous to *Arabidopsis thaliana* stress-related genes, having no expression in bold-seeded genotype and significantly expressed in small-seeded phenotype at 0 DAA. The putative functions are indicated.

**Transcript ID**	**Putative gene**
c26166_g1_i1_AT1G05260	Rare cold inducible 3
c28789_g2_i1_AT1G14870	Plant cadmium resistance2
c20513_g1_i1_AT1G19610	Low-molecular-weight cysteine-rich 78
c23044_g1_i1_AT1G59620	CW9
c40690_g1_i2_AT2G14610	Pathogenesis-related gene 1
c37541_g1_i1_AT2G16060	Class I hemoglobin
c38038_g1_i1_AT3G04720	Pathogenesis-related 4
c38038_g1_i2_AT3G04720	
c37205_g1_i1_AT3G04720	
c37185_g1_i3_AT4G16940	Disease resistance protein
c39524_g1_i1_AT4G21440	MYB-like 102
c39524_g2_i1_AT4G21440	
c43475_g1_i3_AT5G45050	WRKY 16
c37828_g1_i1_AT5G13080	WRKY 75
c37828_g1_i2_AT5G13080	
c31967_g1_i1_AT5G13080	
c24957_g1_i1_AT5G45710	Heat shock transcription factor A4C
c35647_g4_i2_AT5G56550	Oxidative stress 3
c35647_g4_i1_AT5G56550	

SupplementaryTable S5. *Arabidopsis thaliana* seed development related genes that show homology to differentially expressed transcripts between transcriptomes of small (HS)- and bold (HC)- seeded litchi genotypes.

**Gene ID**	**Putative gene**
AT3G01610	AAA-typeATPase (CD48C)
AT1G01370	Centromere specific histone (H3 variant)
AT2G32950	E3 ubiquitin – protein ligase (COP1)
AT1G12260	NAM-like protein
AT5G49010	DNA replication, GINS complex (SLD5)
AT5G15920	SMC family protein (MSS2)
AT5G13690	Alpha-N-acetyl-glucosaminidase (NAGLU/ CYL1)
AT3G48110	Glycine tRNA ligase
AT5G27740	DNA replication factor (RFC3)
AT1G08840	Helicases/nuclease (Dna2)
AT3G17300	Unknown
AT3G20070	Unknown
AT5G07280	LRR receptor kinase
AT1G67320	DNA polymerase alpha (POLA3)
AT3G06350	Dehydroquinatedehydratase; Shikimate dehydrogenase
AT2G44190	Novel microtubule associated protein
AT5G18700	Microtubule associated kinase (RUNKEL)
AT2G45690	Peroxisomal biogenesis factor (Pex16)
AT5G16715	Embryo defective 2247
AT1G64790	Translational activator(GCN1)
AT3G54720	Putative glutamate carboxypeptidase (Altered meristem program 1)
AT5G67570	PPR protein
AT5G48600	Condensin (Structural maintenance chromosomes protein 4)
AT2G01190	Uncertain
AT3G50870	GATA factor transcriptional regulator
AT1G76620	Unknown

AT4G02060	DNA replication licensing factor
AT2G34650	Serine threonine protein kinase
AT1G08560	Cytokinesie specific syntaxin
AT5G13480	Nuclear RNA binding protein
AT1G44900	Putative DNA helicase subunit (MMC2)
AT1G66520	Methionyl-tRNAformyltransferase
AT5G62410	SMC2 condensin
AT5G49160	Methyltransferase
AT1G71720	RNA binding protein
AT3G54650	F box protein
AT1G78580	Trehalose-6-phosphate synthase 1
AT1G67730	B-ketoacyl-coenzyme A reductase
AT3G06350	Dehydroquinate - Shikimate dehydrogenase

Supplementary Table S6. Putative function of differentially expressed transcripts selected for validation of relative expression analyzed from RNA-seq libraries, by real time PCR. Functions of the annotated genes were assigned on the basis of homology with the genome database of *Arabidopsis thaliana*.

**Transcript ID**	**Homologous genes TAIR ID**	**Putative gene**
c35336_g1_i2	AT2G29420	Glutathione S-transferase 25
c35680_g1_i1	NA	NA
c36844_g1_i1	AT2G32150	Haloacid dehalogenase-like hydrolase (HAD) superfamily protein
c41239_g1_i1	AT1G68320	MYB domain protein 62
c41297_g1_i1	AT4G11650	Osmotin 34
c42714_g3_i1	NA	NA
c47655_g1_i1	NA	NA
c47849_g1_i1	NA	NA
c50527_g1_i1	NA	NA
c57835_g1_i1	NA	NA

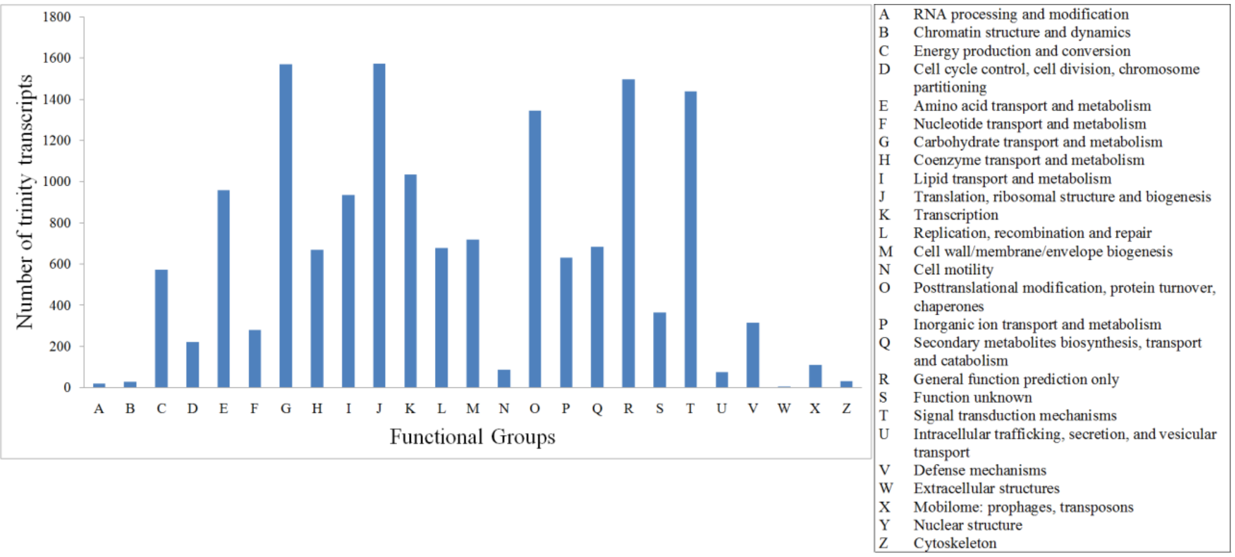

Supplementary Figure S1. Transcripts classified as Clusters of orthologous Groups (COG). All transcripts were aligned to the COG database to predict and classify possible functions. A total of 15,840 transcripts were classified into 25 groups.

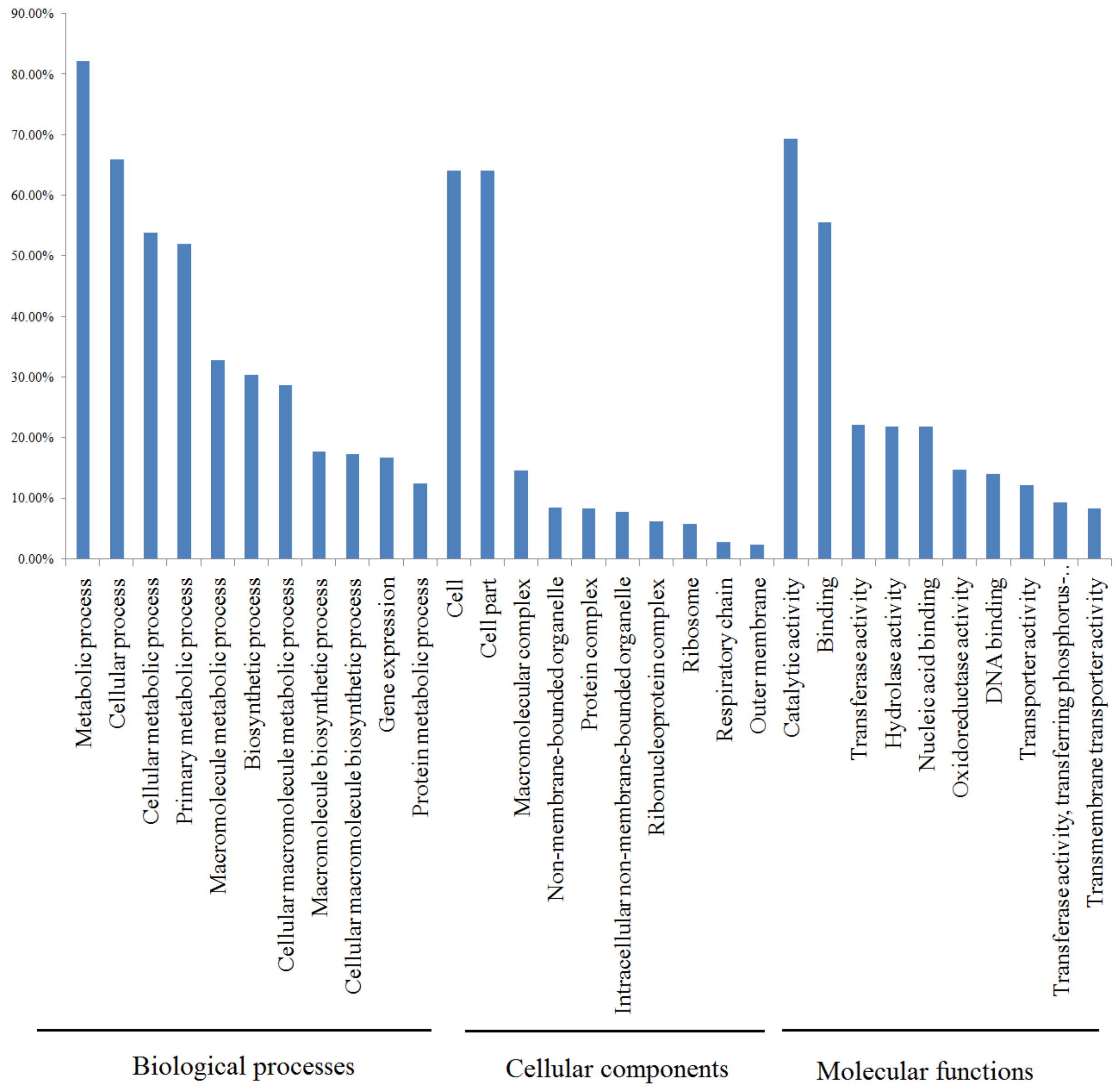

Supplementary Figure S2. Functional classification of assembled transcripts based on Gene Ontology (GO). The results are summarized in three main GO categories: biological processes, cellular components and molecular functions. The x-axis indicates the subcategories of GO terms and y-axis represents % of transcripts belonging to the GO term.

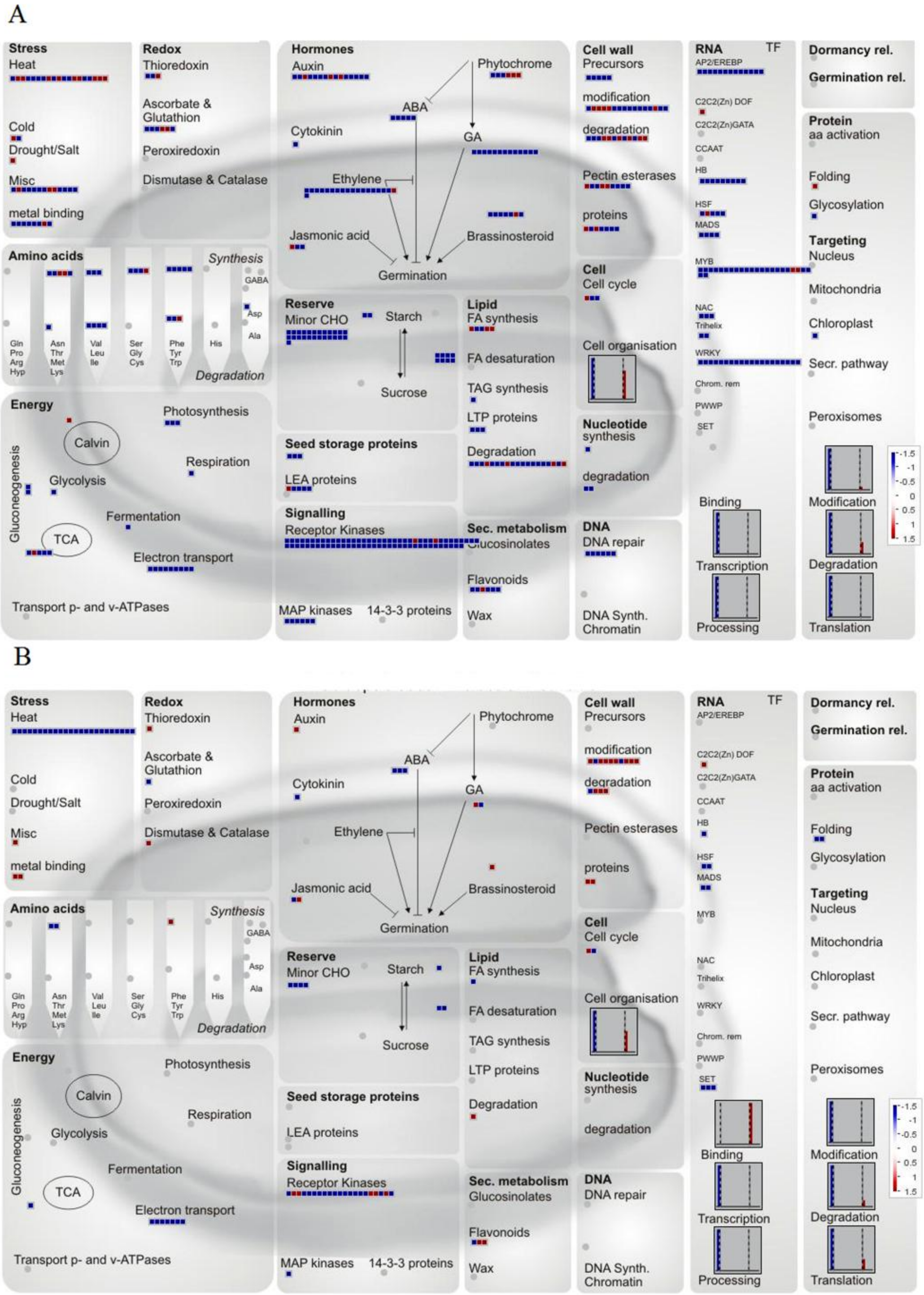

Supplementary Figure S3. MapMan display of differentially expressed putative genes regulating litchi ovule development in transition from 6 DAA to 14 DAA. Fold change (14DAA vs 6 DAA) is plotted for A) bold-seeded (HC), and B) small seeded (HS) ovules of litchi.

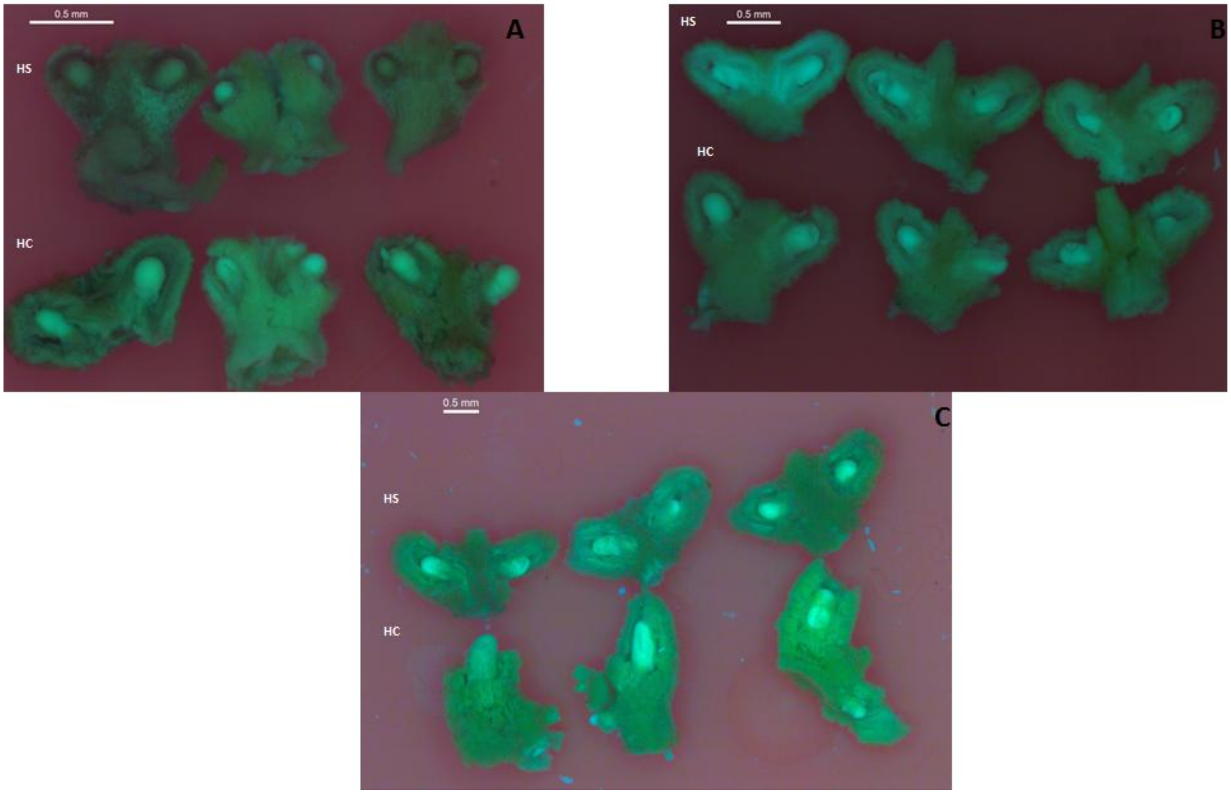

Supplementary Figure S4. Dissected developing fruits of small- (HS) and bold seeded (HC) litchi at (A) 0 DAA B) 6 DAA and C) 14 DAA.

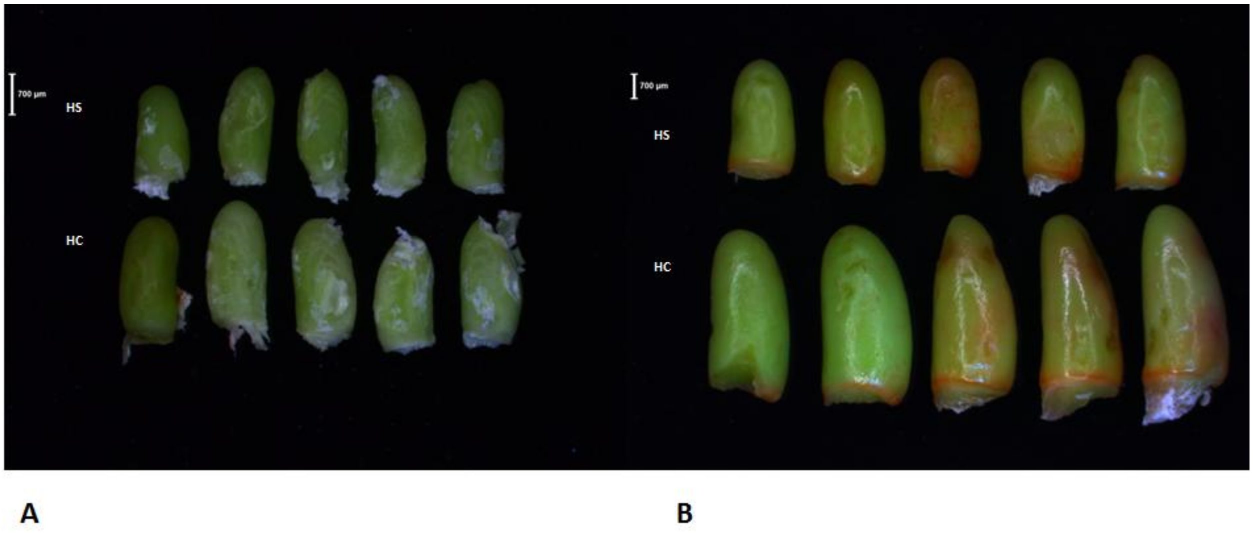

Supplementary Figure S5. Developing ovules of small- (HS) and bold-seeded (HC) litchi at A) 6 DAA and B) 14 DAA.

Supplementary Data 1. Differentially expressed transcripts ($\log_2$ fold ≥ 2 ; *P*-value ≤ 0.001) in ovules, bold- vs small-seeded litchi.

Supplementary Data 2. Differentially expressed transcripts ($\log_2$ fold ≥ 2; *P*-value ≤ 0.001) at different developmental stages of ovules (0 vs 6, 6 vs 14 DAA) in litchi.

Supplementary Data 3. Expression pattern of differentially expressed ($\log_2$ fold ≥ 2; *P*-value ≤ 0.001) hormone-related genes at early ovule developmental stages of bold-seeded (HC) vs small-seeded (HS) litchi genotypes at three developmental stages. FPKM of putative auxin, brassinosteroid biosynthesis and auxin transport transcripts.

Supplementary Data 4. Expression pattern of differentially expressed ($\log_2$ fold ≥ 2; *P*-value $\leq$ 0.001) putative transcription factors at early ovule developmental stages of bold-seeded (HC) vs

small-seeded (HS) litchi genotypes at three developmental stages. FPKM of transcription factor related putative transcripts.

Supplementary Data 5. Gene specific primers used in real-time PCR analysis to validate expression level of putative genes in the developing ovules of litchi (HS and HC).
